# Gastric Necrosis: A Rare Complication of Gastric Mucormycosis

**DOI:** 10.7759/cureus.35810

**Published:** 2023-03-06

**Authors:** Chetna Rathi, Raju K Shinde, Sajika P Dighe, Yashwant Lamture, Rajat Mahawar

**Affiliations:** 1 General Surgery, Jawaharlal Nehru Medical College, Datta Meghe Institute of Higher Education and Research, Wardha, IND

**Keywords:** invasive fungal infection, acute pancreatitis, gastrointestinal mucormycosis, mucormycosis, gastro-colic fistula, gastric necrosis

## Abstract

Mucormycosis is an invasive fungal infection. The prevalence is low for this disease, and the most common site of its occurrence in the gastrointestinal system is the stomach. The clinical signs and symptoms of gastric mucormycosis are vague such as pain in the abdomen, nausea, vomiting, haematemesis, etc. In the current study, a 42-year-old male patient came with complaints of pain in the abdomen associated with vomiting and mild-grade fever based on vague clinical presentation and imaging like upper gastrointestinal endoscopy and computer tomography angiography of the abdomen. Our preoperative diagnosis was gastrocolic fistula secondary to acute on chronic pancreatitis, which was managed by emergency exploratory laparotomy, resection, and anastomosis. Histopathological examination was successful in confirming the diagnosis of mucormycosis. Through this case report, we intend to draw surgeons' and physicians' attention to gastrointestinal mucormycosis, an emerging cause of gastric necrosis in young patients in the post-coronavirus disease era, and that physicians need to be more aware of the consequent high mortality and morbidity. Early diagnosis followed by aggressive debridement, antifungal therapy, and managing the underlying disease is the most efficient way to reduce mortality associated with the disease.

## Introduction

The estimated prevalence of mucormycosis in India is 70% higher than global data; out of these, 2-8% accounts for gastrointestinal mucormycosis [1]. Mucormycosis is caused by the fungi Mucoromycotina, order Mucorales [2]. Diabetes mellitus is the most common risk factor in adults. In the current case study, the patient presented with pain in the abdomen associated with vomiting. High-risk for invasive mucormycosis include the conditions of diabetes mellitus, immunocompromised and post-organ transplantation [3-6]. The disease is found to most commonly affect the rhino-orbital-cerebral or pulmonary region. An obvious rise in cases of gastrointestinal mucormycosis has been noted since; a search for the title words "gastric" or "gastrointestinal" and "mucormycosis" or "zygomycosis" revealed 23 publications from 1990-1999, 50 from 2000-2011, and 90 from 2012-2022. Due to its short course and vague symptoms, it is often diagnosed post-mortem based on histopathological examination. Thus, our pre-operative diagnosis was pancreatitis-induced gastrocolic fistula.

## Case presentation

A 42-year-old chronic alcoholic male presented to the emergency room with the chief complaint of pain in the abdomen and vomiting for 12 days. Abdominal pain was sudden and excruciating, localizing in the epigastric region and radiating to the back. It was relieved by lying in the prone position and by medications. Vomiting that followed meals was non-bilious, non-projectile, with contents such as food and water. It was associated with mild-grade fever. The patient had no other significant medical history. Abdominal and digital rectal examination revealed no significant findings.

**Figure 1:**
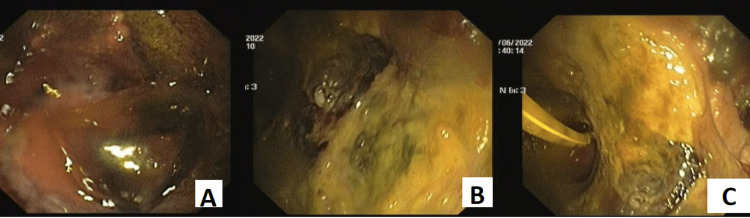
Upper gastrointestinal endoscopy A: first upper gastrointestinal endoscopy shows inflamed gastric mucosa with an ulcer and concealed perforation along the anteroinferior wall of the stomach. B and C: second upper gastrointestinal endoscopy shows necroed gastric mucosa and the nasogastric tube is visible.

On admission, a routine blood check was performed, showing leukocytosis (white blood count of 21,500 cells/mm^3^), deranged coagulation profile (international normalized ratio of 1.32), raised lipase (326 U/L), hyperglycemia (random blood sugar [RBS] of 243), glycosylated hemoglobin (HbA1c) of 8.65%, hyponatremia of 128 mmol/L. On further evaluation with ultrasound and computer tomography of the abdomen and pelvis, a definitive diagnosis of emphysematous gastritis with a cystic lesion in the head of the pancreas, most likely a pseudocyst of size 4x4 cm, and associated bilateral lung consolidation was made. In view of emphysematous gastritis to rule out gastric necrosis, an upper gastrointestinal endoscopy was performed, which showed diffuse ulceration in the body, antrum, and pylorus of the stomach with a bulge seen at the body of the stomach (Figure 1). Following this, the patient continued symptomatic treatment and had symptomatic relief.

After three days, the patient had an episode of hematemesis, for which we conducted an emergency upper gastrointestinal endoscopy (UGIE) and computer tomography angiography (CTA) of the abdomen. They concurrently showed significant thinning with concealed perforation from the anteroinferior stomach wall, along with acute pancreatitis with an area of necrosis within the pancreas and peripancreatic fluid (Balthazar score of 6/10). There was evidence of emphysematous gastritis, with the stomach showing adhesions to the pancreas and small pockets of air in the peritoneal cavity. After angiography, there was a small aneurysm arising from the gastroduodenal artery, which was embolised on the same day and was a suspected cause of hematemesis. Assuming a provisional diagnosis of a complicated case of acute on chronic pancreatitis with hematemesis, the conservative line of management with a nasojejunal tube, intravenous fluids, and enteral nutrition was continued.

With this approach, the patient improved over seven days but suddenly had a novel complaint of melena for which prophylactic UGIE and colonoscopy were performed. UGIE revealed similar findings as before. Colonoscopy successfully led us to diagnose a gastrocolic fistula along with small bowel communication with walled-off pancreatic necrosis (WOPN; seeFigure 2). It revealed a defect in the transverse colon with normal-looking mucosa around it. The necrotic cavity was in communication with the stomach, small bowel, and colon. In-situ nasojejunal tube and WOPN were also evident. At this site, a visible vessel with a blood clot was identified, which seemed to be the likely source of the bleeding. For this reason, an adrenaline injection was administered around the source of bleeding, and the vessel was hemoclipped endoscopically.

**Figure 2:**
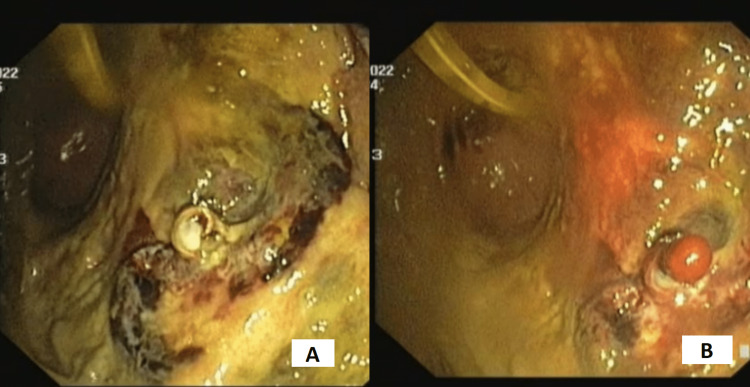
Colonoscopy showing gastrocolic fistula along the nasogastric tube in the transverse colon A: shows necrosis in the transverse colon. B: confirms the presence of gastrocolic fistula by the presence of the nasojejunal tube visualized during colonoscopy in the transverse colon.

In view of a gastro-colic fistula secondary to acute on chronic pancreatitis, we planned exploratory laparotomy. Intra-operatively, the necrotic part of the pancreas was identified. Distal two-thirds of the stomach was found to be necrosed; only the fundus and pyloric end were spared. Two rents in the transverse colon and one rent in the small bowel at the duodenojejunal junction were evident due to necrosis. Pus collection was noted in the para-duodenal area. The left lobe of the liver was found to be necrosed.

**Figure 3:**
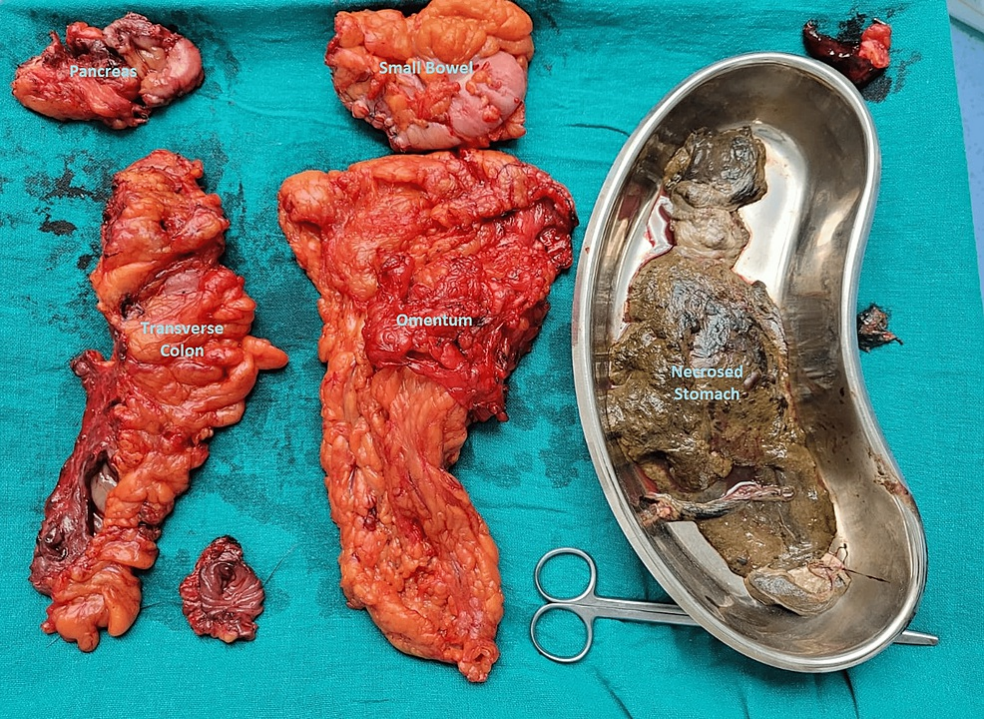
Specimen photo

Hence, the patient underwent a necrosectomy for necrosed part of the stomach, wall of the pseudocyst, liver, and pancreas. Gastrointestinal continuity was maintained with duodenal stump closure, jejunojejunostomy, and gastro-jejunostomy (Bilroth-2), followed by colo-colic anastomosis (Figure 3). Intra-operatively, the patient had an episode of hypotension and was transfused with three units of packed red cells, one unit of whole blood, and eight units of fresh frozen plasma.

**Figure 4:**
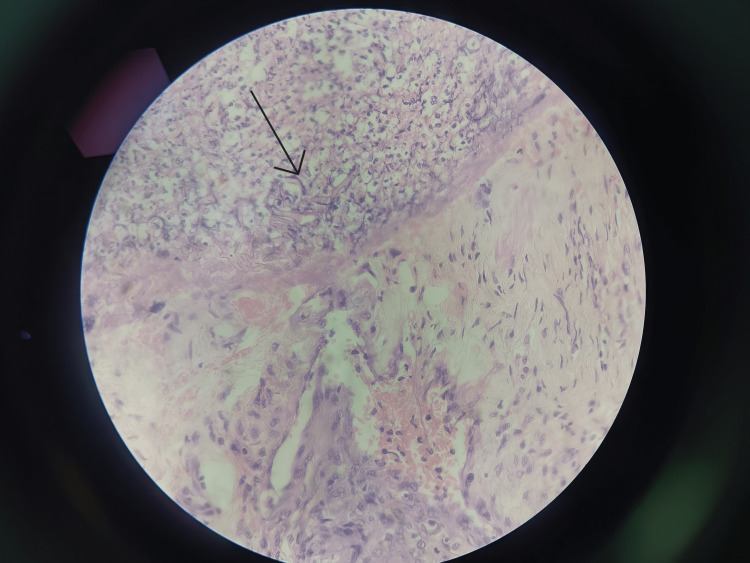
Histopathological Image showing hyphae (arrow)

Postoperatively, the patient was managed in the intensive care unit by a multi-disciplinary team comprising Intensivists, surgeons, and a gastroenterologist. On postoperative day two, the patient experienced anuria which led to acute renal failure. His renal system was managed aggressively, but his health eventually deteriorated due to progressive acute kidney injury, sepsis, multi-organ dysfunction, and hypotension. On postoperative day three, the patient went into multiple organ dysfunction syndrome and, despite all the possible efforts, succumbed to sudden cardiac arrest. The histopathological examination confirmed the diagnosis of mucormycosis and gangrenous necrosis of the stomach and excised portion of the jejunum with inflammation and necrosis of the transverse colon and omentum was made (Figure 4). Specimen labeled as the part pancreas and liver showed necrosis.

## Discussion

Mucormycosis or zygomycosis is an angioinvasive fungi known to cause lethal opportunistic infection [4]. The fungal infection occurs upon inhalation of sporangiospores or directly via inoculation over ulcers on the skin or mucosa in immunocompromised people [5]. It has been shown to affect the pulmonary, rhino-cerebral, gastrointestinal, cutaneous, or central nervous systems. In the case of the involvement of two or more non-contiguous organ systems, it is considered to be disseminated and highly invasive. Disseminated mucormycosis commonly affects the tissues of the lungs and brain, with the infection usually originating in the lungs [7]. Pulmonary and rhinocerebral forms are the most common forms of infection, while primary gastrointestinal mucormycosis is very rare.

Though the whole of the alimentary tract is susceptible to mucormycosis infection, the stomach is most commonly affected, followed by the colon and ileum. This infection occurs following the ingestion of infected sputum or secondary colonization of pre-existing ulcers. Clinically, it presents with non-specific symptoms, fever, gastrointestinal bleeding, and perforation. In premature neonates, gastrointestinal mucormycosis infection may present as necrotizing enterocolitis [1]. Mucormycosis that affects only the stomach, or gastric mucormycosis, exists in one of three forms: colonization, infiltration, and vascular invasion. While there have been previously published case reports of gastric mucormycosis in premature neonates and malnourished children, primary gastric mucormycosis in adults, although rare, appears to be on the rise [8].

Gastric mucormycosis is diagnosed based on histopathology showing aseptate, broad periodic acid-Schiff (PAS)-positive fungal hyphae adjacent to the necrotic areas, though the organism, genus, and species are identified only on culture and are aimed for epidemiological, therapeutic, and prognostic implications.

Amphotericin B, a polyene antifungal agent, is highly effective against fungal infection. Though for a complete cure, combining an antifungal agent with debulking surgery to remove all infected tissue is necessary. Roden et al., in their review, have confirmed the significance of multimodality treatment with surgery and amphotericin B [3]. To prevent mortality and strong clinical suspicion, diagnosed and combined therapy with aggressive surgical debridement and amphotericin B therapy are required.

Gastric mucormycosis is rare and difficult to diagnose pre-operatively. In this post-coronavirus era, there have been more cases of mucormycosis. Therefore, this demands attention on early diagnosis followed by aggressive management of invasive fungal infection.

## Conclusions

The era of invasive fungal infections seems to be on the rise. Though rare, the incidence of mucormycosis has seen a significant rising trend. Hence, there is an urgent need to direct the focus on new techniques and methods for early diagnosis and management of complications secondary to invasive fungal diseases. The above case report demonstrates that despite the rigorous effort through multiple upper gastrointestinal endoscopies, we failed to diagnose the disease and manage it efficiently. 
